# Vapor-etching honeycomb-like zinc plating layer for constructing anti-corrosion lubricant-infused surfaces

**DOI:** 10.3389/fchem.2023.1273674

**Published:** 2023-09-28

**Authors:** Xiaorui Song, Na Li, Zhongshan Wang, Shuangjian Li, Yuanyuan Hou

**Affiliations:** ^1^ Centre for Advanced Laser Manufacturing (CALM), School of Mechanical Engineering, Shandong University of Technology, Zibo, China; ^2^ Department of Stomatology, Zibo Maternal and Child Health Hospital, Zibo, China; ^3^ Institute of New Materials, Guangdong Academy of Sciences, National Engineering Laboratory of Modern Materials Surface Engineering Technology, Guangdong, China

**Keywords:** superhydrophobic, lubricant, electroplate, honeycomb-like, anticorrosion

## Abstract

**Introduction:** Biomimetic lubricant-infused porous surfaces are developed and applied for omniphobicity and corrosion protection, which exhibit great advantages compared to superhydrophobic surfaces.

**Methods:** Herein, superhydrophobic Fe@E-Zn@PFOA was prepared via the electrodeposition of laminated Zinc coating, further vapor etching, and post-modification with perfluoro caprylic acid. The facile, inexpensive, and environment-friendly water vapor etching process can form a porous honeycomb-like structure. Moreover, the perfluoropolyether lubricant was wicked into the porous and superhydrophobic surfaces, obtaining lubricant-infused surfaces of Fe@E-Zn@PFOA@PFPE.

**Results and discussion:** The influences of the textured roughness and chemical composition on the surface wettability were systematically investigated. The Fe@E-Zn@PFOA@PFPE performs omniphobicity with small sliding angles and superior corrosion resistance compared with the superhydrophobic surface, owing to their multiple barriers, including infused lubricant, hydrophobic monolayers, and compact Zn electroplating coating. Thus, the proposed lubricant-infused surface may provide insights into constructing protective coatings for the potential applications of engineering metal materials.

## 1 Introduction

Corrosion of metal materials is unavoidable in daily production and life; it causes significant wastage of resources and energy and even leads to potential safety issues (Zhang et al., 2018; Wang and Cao, 2023; Wang et al., 2023). How to improve the corrosion resistance of metal materials is an important research topic in the field of engineering equipment, materials, and advanced manufacturing. Recently, various techniques, including anodic oxidation, cathodic protection, electroplating, and conversion coatings, which can endow the metal substrate with a barrier layer, have been broadly introduced to protect metal from corrosion (Hou et al., 2020; Singh et al., 2023; Zehra et al., 2023). Among these techniques, Superhydrophobic coating has been considered one of the most straightforward and effective strategies (Zhang and Xu, 2021).

In general, superhydrophobic coatings are commonly constructed by introducing low-surface-energy modifiers on rough structures or textured rough structures on low-surface-energy materials (Zhang et al., 2021). For the superhydrophobic surface, hydrophobic air could be easily absorbed into the rough structure to form an air cushion that acts as a protection layer, forming the Cassie-Baxter wetting state. Thus, the hydrophobic coating can minimize the contact area between the underlying substrates and foreign corrosive liquids, which is conducive to reducing the problem of corrosion (Simpson et al., 2015; Qian et al., 2017; Vazirinasab et al., 2018).

Nevertheless, there are several drawbacks of using superhydrophobic coatings for anti-corrosion, which greatly restrict their practical applications. At first, the formed air cushion in the hydrophobic rough structure is thermodynamically unstable and is prone to be depleted, resulting in the loss of the air layer and exposing the area between the coating and the corrosion liquids (Verho et al., 2011; Lu et al., 2015). In addition, most air-trapped micro/nano-hierarchical structures are prone to be destroyed or collapsed to generate massive fractured cracks by localized damages, such as vibration, impact, or thermal shock (Wang et al., 2020). The broken structure could serve as a penetration path of corrosive ions, which further results in the exposure of the metallic surface to the outer corrosive environment. Therefore, it is vital to exploit an alternative stable coating to protect the substrate from corrosion (Tian et al., 2016).

Inspired by the Nepenthes pitcher plant, Aizenberg et al. (Wong et al., 2011) prepared a slippery liquid-infused porous surface (SLIPS) by infusing the lubricant into the micro-nano porous structure. To date, there are various porous structures including a pit array structure (Kamei and Yabu, 2015), trench array structure (Yong et al., 2017), columnar array structure (Rykaczewski et al., 2014), swelling polymers (Zhu et al., 2019), pleated structure (Gao et al., 2021), and particle stacking porous structure (Lou et al., 2020), which have been developed and fabricated by laser ablation texture, physical deposition technology, corrosive medium etching, or coating methods (Chen et al., 2020). Compared to the gaseous barrier layer of the superhydrophobic surface, the SLIPSs obtained by liquid-infused technology are more stable with metastable states (Zhang et al., 2014; Liu et al., 2022). Thus, the SLIPSs with stable lubricant layers can withstand harsh external circumstances and further exhibit superior liquid repellency, pressure stability, and self-healing properties (Wexler et al., 2015; Liu et al., 2018; Yu et al., 2020). By virtue of the excellent omniphobicity of the infused lubricant layer, the SLIPSs have been developed to protect the original metal substrates from corrosion (Ding et al., 2023; Lv et al., 2023; Qin et al., 2023; Tripathi et al., 2023; Yan et al., 2023). Tu et al. (Zhang et al., 2017) designed a double-layered SLIPS coating to protect the AZ31 Mg alloy from corrosion and icing. Choi et al. (Lee et al., 2017) impregnated oil into the hydrophobic nanoporous AAO layer to enhance the corrosion resistance of an AAO layer by two and four orders of magnitude compared to that of a superhydrophobic AAO. Li et al. (Xiang et al., 2018) used a facile electroplating method combined with chemical replacement to fabricate the SLIPS, which is suitable for any metal corrosion protection. Despite these great achievements, these SLIPSs are always required to fabricate hierarchical porous structures, which involves a complicated hole-making process as well as the vulnerability of the surfaces (Vorobev, 2014; Sett et al., 2017). Moreover, most of the interconnected porous structures used as oil storage sites are easily destroyed under hydrodynamic shear, resulting in the loss of lubricating oil (Howell et al., 2015; Meng et al., 2018). Therefore, it is highly desired to exploit a strategy for fabricating robust SLIPSs in a facile, inexpensive, and environmentally friendly way.

In this study, we first prepared laminated Zinc coating on an Fe surface via electrodeposition in deep eutectic solvent. Furthermore, the prepared Zn coating (Fe@Zn) was selectively etched by water vapor to form a massive interconnected honeycomb-like porous structure without using any corrosive medium or expensive equipment. Moreover, the obtained Fe@E-Zn surface was post-modified with perfluoro caprylic acid (PFOA) to prepare the superhydrophobic Fe@E-Zn@PFOA surface. Further infusing the perfluoropolyether lubricant into the superhydrophobic porous surface can prepare the lubricant-infused surface of Fe@E-Zn@PFOA@PFPE. The wettability and anti-corrosion properties of different samples were systematically investigated. The proposed lubricant-infused surface was shown to have great potential applications in engineering metal material protection.

## 2 Materials and methods

### 2.1 Materials

Fe plates and Zinc plates of the same size, 30 mm × 40 mm, were purchased commercially. Choline chloride (HOCH_2_CH_2_(CH_3_)_3_N^+^Cl^−^, ChCl), ethylene glycol (HOCH_2_CH_2_OH, EG), ZnCl_2_•6H_2_O, and perfluorooctanoic acid (PFOA) were analytical grade and supplied by Sinapharm Chemical Reagent Co., Ltd. The perfluoropolyether lubricant (PFPE, Krytox GPL 103) was supplied by DuPont Chemours Company. Deionized water and ethyl alcohol were used throughout the experiment. All other chemical reagents were analytical grade and used without further purification.

### 2.2 Preparation of the Fe@Zn electroplating coating in deep eutectic solvent

Deep eutectic solvent (DES) was fabricated by adding choline chloride (ChCl) into ethylene glycol (EG) with a molar ratio of 1:2 and then stirring at 70 °C for 2 h. Then, 13.63 g ZnCl_2_•6H_2_O particles were put into 200 mL ChCl:2EG DES solution. The electrolyte solution was obtained until the complete dissolution of particles.

The electroplating Zn coating was carried out in a three-electrode system, in which the zinc plate (Zn, 30 × 40 mm), Fe plate (Fe, 30 × 40 mm), and saturated calomel electrode were played as working electrode, counter electrode, and reference electrode, respectively. The electrodeposition of Zn was performed with a constant voltage of −1.50 V at 40 °C for 2 h, which was named Fe@Zn sample.

### 2.3 Construction of the superhydrophobic surface and lubricant-infused surfaces

The prepared Fe@Zn was etched with water vapor using a homemade humidifier (humidity was 75%) at an ambient temperature for 2, 4, 6, and 8 h, obtaining Fe@E-Zn-X h, in which X was the etching time. For convenience, the Fe@E-Zn-6h was abbreviated as Fe@E-Zn. Subsequently, the etched Fe@E-Zn was immersed in 30 mL 0.05 M PFOA solution (ethanol) for 10 h and obtained the superhydrophobic Fe@E-Zn@ PFOA. Furthermore, 2 mL perfluorinated lubricant PFPE was dropped onto the Fe@E-Zn@PFOA, and the lubricant could rapidly infiltrate into the honeycomb structure via capillary effect. Then the sample was vertically placed for 10 min to wipe out excess oil by gravity-driven drainage, forming the lubricant-infused surface of Fe@E-Zn@PFOA@PFPE.

### 2.4 Measurements and characterization

The surface structure and element distribution of the samples were watched by scanning electron microscope (SEM, Hitachi su1510, Hitachi Co., Japan) with energy dispersive spectrum (EDS). The chemical compositions of samples were measured by X-ray photoelectron spectroscopy (XPS, Thermo Scientific ESCALAB 250Xi). The functional groups and crystal structure of the specimen were characterized by Fourier transform infrared spectroscopy (FTIR, Thermo Fisher Scientific iS50) and X-ray diffraction (XRD, Bruker D2 PHASER), respectively. The contact angle (CA) and the sliding angle (SA) of different droplets (10 μL) onto samples were tested using the JC 2000D1 contact angle meter (Zhongchen digital equipment Co., Ltd.). The average CA and SA values were obtained by measuring the same sample in three different positions. The corrosion resistance of the samples was measured by electrochemical workstation (CHI660E, Shanghai Chenhua Instrument Co., Ltd.).

## 3 Results and discussion

### 3.1 Morphology and chemical composition of the samples

**FIGURE 1:**
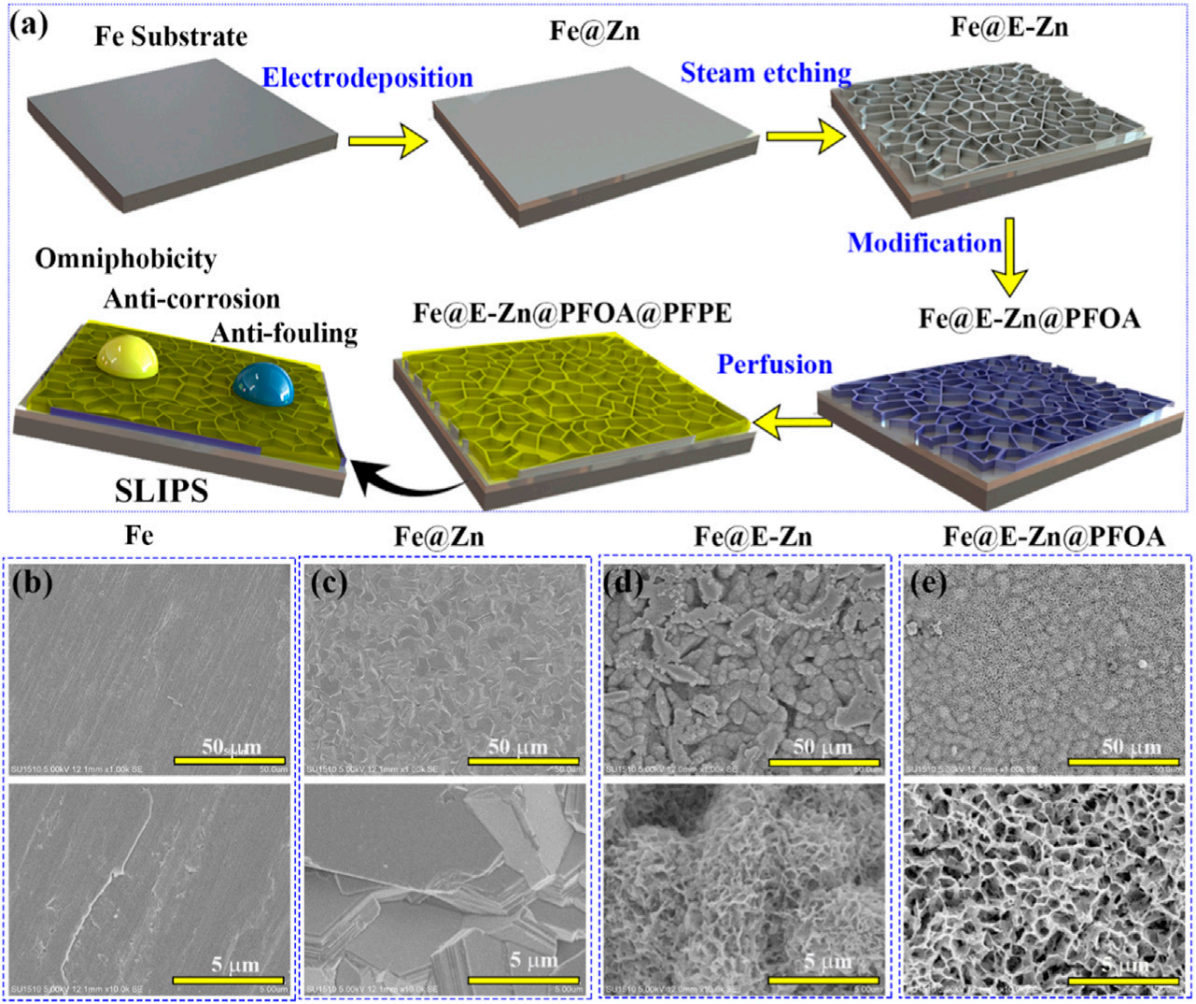
**(A)** Schematic diagram of the fabrication process of Fe@E-Zn@PFOA@PFPE. **(B–E)** SEM images Fe **(B)**, Fe@Zn **(C)**, Fe@E-Zn **(D)**, and Fe@E-Zn@PFOA **(E)**.

**FIGURE 2:**
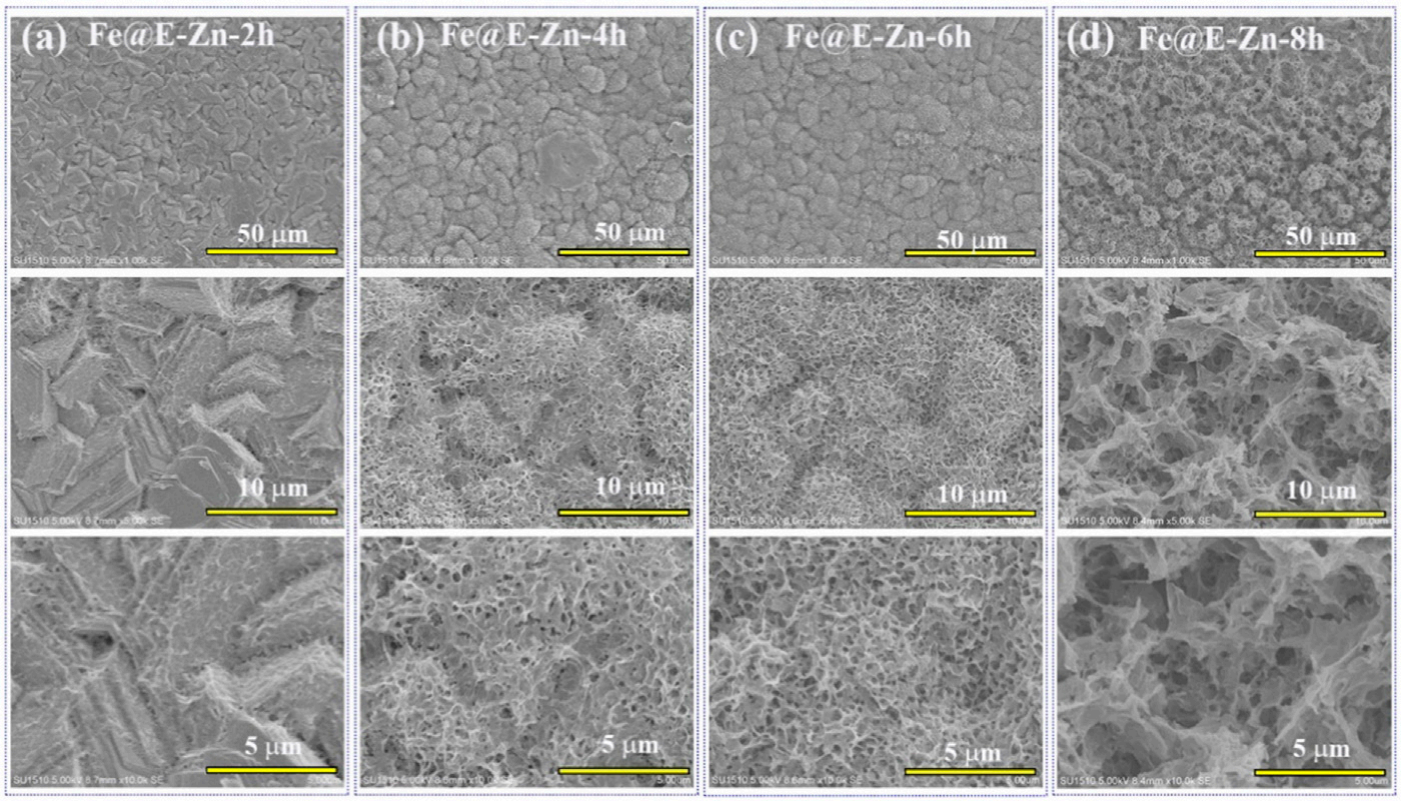
SEM images of the Fe@E-Zn after vapor-etching for different times, including 2 h **(A)**, 4 h **(B)**, 6 h **(C)**, and 8 h **(D)**.

Figure 1A presents the fabrication process of the lubricant-infused surface of Fe@E-Zn@PFOA@PFPE. The original Fe plate is relatively smooth, with several trenches and convex hulls as shown in Figure 1B. After the electrodeposition process, the stacked Zn nanosheet layers were tightly coated on the metallic Fe@Zn surface in the ChCl:2EG DES (Figure 1C). Notably, the Zn plating coating in DES can avoid the hydrogen evolution that frequently occurred in the water-based electroplating solution and form the steady stacked structure [27, 28]. Then, the Fe@Zn was vertically placed against the water vapor in a sealed box at different times. As shown in Figures 2A–D, the Zn coating was gradually etched to form the porous structure with increasing etching time.

After etching for 2 h, the exposure positions of the Fe@E-Zn−2 h with the two-dimensional layer were etched to form rough grooves (Figure 2A). Then, after etching for 4 h, a large number of unconnected macro-porous structures appeared (Figure 2B). However, the hole structure is not interconnected. Further increasing the etching time to 6 h, the Zn coating was etched to form a dense honeycomb porous structure (Figure 2C). As for the Fe@E-Zn-8h, the honeycomb structure was completely destroyed and formed massive recessed holes (Figure 2D). Notably, the position of the surface also influences the etching effect. As shown in Supplementary Figure S1, after etching for 6h, the back surface of the samples generated a larger number of needle-like structures, which is greatly different from the front of the Fe@E-Zn-6h. Based on the consideration of the stability of the oil storage structure, the Fe@E-Zn-6h was finally chosen for further investigation. Moreover, the vapor-etched Fe@E-Zn with the massive hydroxy group could react with PFOA by the silane shrinkage condensation (Figure 1D). After modification by PFOA, the structure of the obtained Fe@E-Zn@PFOA did not change (Figure 1E). Moreover, PFPE was infused into the Fe@E-Zn@PFOA via the capillary forces and Van der Waals force between the PFOA and PFPE.

**FIGURE 3:**
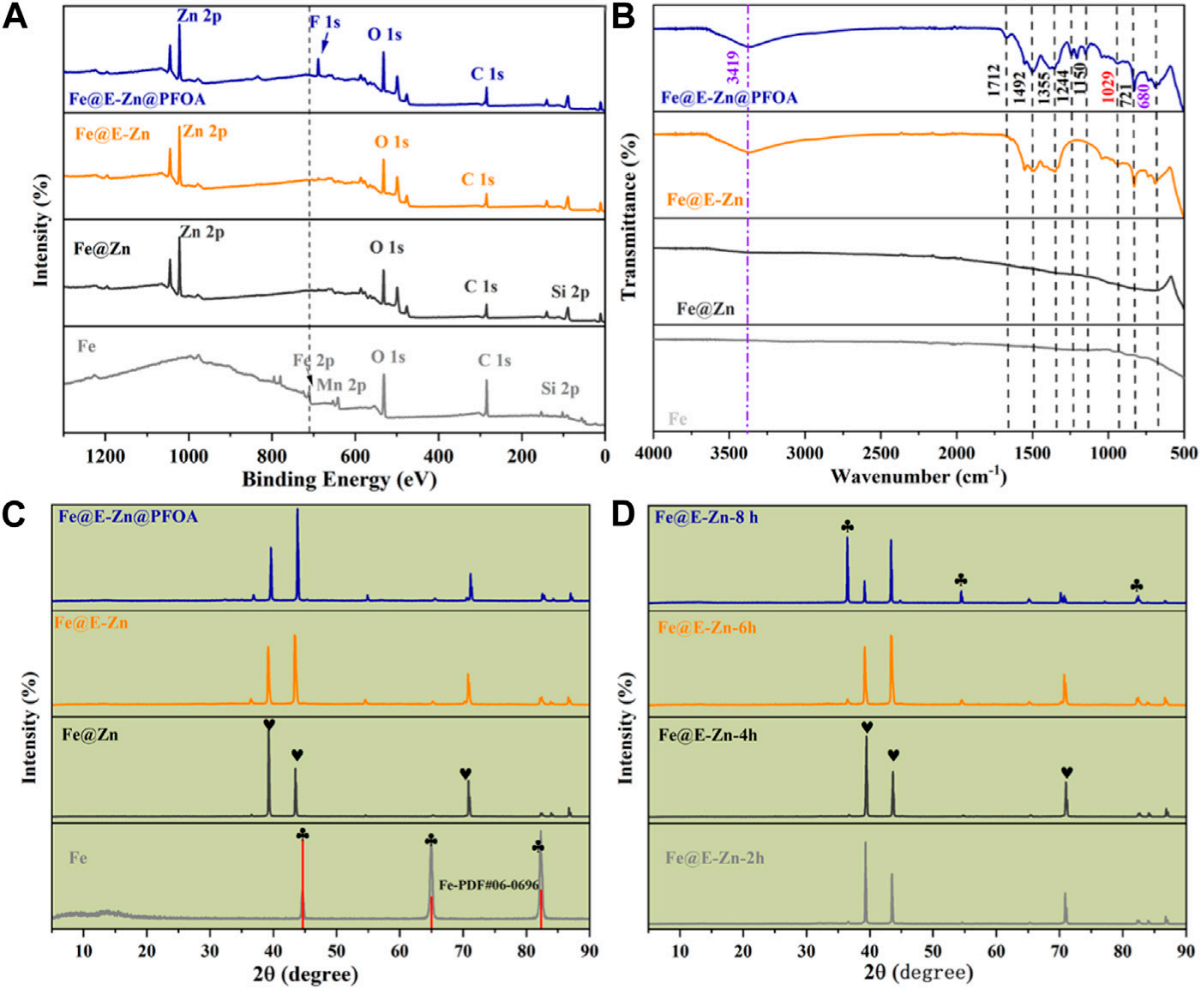
Full XPS spectra **(A)** and FTIR spectra **(B)** of different samples. **(C)** XRD spectra of Fe@E-Zn after vapor-etching for different times, **(D)** XRD spectra of different samples.

Furthermore, the elemental compositions and distributions of the specimen were measured by the XPS spectra and EDS mapping. As demonstrated in Figure 3A, the pristine Fe plate shows its typical. The peaks of 712.4 eV, 642.5 eV, 530.4 eV, and 283.4 eV belong to pristine Fe plate, which is attributed to Fe 2p, Mn 2p, O 1s, and C 1s, respectively. For the Fe@Zn samples, the newly appearing peaks at 1044.7 eV, 1019.4 eV, and 498.7 eV are ascribed to Zn 2p and Zn auger, respectively. Meanwhile, the peak at 712.4 eV corresponding to the Fe element vanished, indicating that the Zn coating completely covered the Fe plate. For the Fe@E-Zn, there are no apparent changes compared to Fe@Zn. After modification with PFOA, the new peak at 690.7 eV ascribed to F 1s appeared and the intensity of the C 1s of the Fe@E-Zn@PFOA greatly improved, simultaneously verifying the successful post-modification with PFOA. Moreover, the element distribution of different samples was tested by the EDS spectra, as shown in Supplementary Figures S2–S5. Supplementary Figure S2 shows that a pure Fe plate has three typical elements including Fe (81.29%), Cr (17.52%), and C (18.28%). As for the Fe@Zn, the element contents are Fe (1.36%), Zn (97.92%), and C (0.72%), respectively (Supplementary Figure S3). After etching by vapor, the main elements of the obtained Fe@E-Zn are Fe, Zn, C, and O, with a mass ratio of 1.47%, 68.35%, 4.12%, and 26.06%, indicating the formation of the massive hydroxy groups (Supplementary Figure S4). As for the Fe@E-Zn@PFOA, the relative amount of the C, F, and Zn increased to 6.01%, 3.24%, and 69.08%, while the content of Fe and O decreased to 1.54% and 20.03%, respectively (Supplementary Figure S5).

Furthermore, the chemical functional group of the samples was characterized by the FTIR spectra, as demonstrated in Figure 3B. The original Fe and Fe@Zn have no obvious absorption peaks. There further appears a broad peak among 3,700–3,200 cm^−1^ for the Fe@E-Zn, which corresponds to the asymmetrical stretching vibration of hydroxy groups from Zn(OH)_2_. After modification with PFOA, there are abundant absorption peaks. The absorption band at 1712 cm^−1^ is assigned to the vibration peak of the carbonyl groups, and the absorption bands at 1244, 1180, and 1150 cm^−1^ can be attributed to the stretching vibration of the C-F group of PFOA. Thus, the FTIR spectra of the Fe@E-Zn@PFOA also confirmed the introduction of the PFOA.

The crystal structure of the samples was further investigated by XRD, as demonstrated in Figures 3C, D. The diffraction peaks at 44.72°, 65.04°, and 82.32° correspond to the (110), (200), and (211) crystal planes of the body-centered cubic structure α-Fe (JCPDS 06-0696) as shown in Figure 3C. For the Fe@Zn, the diffraction peaks at 38.99°, 43.42°, and 70.80° are attributed to (100), (101), and (103) lattice planes of the Zn coating (JCPDS 04-0831). The Fe@E-Zn exhibited new peaks at 36.08° and 54.32°, which were ascribed to (002) and (102) crystal planes of hexagonal zinc Zn(OH)_2_ (JCPDS 38-0385). Furthermore, the XRD spectra of the Fe@E-Zn with different etching times were tested as demonstrated in Figure 3D. After etching for 2 h and 4 h, the intensity of the peaks at 38.99° and 43.42° was similar to Fe@Zn. After etching for 6 h, the intensity of the 43.42° peak was higher than the 38.99° peak, indicating the selective etching of (100) crystal plane. The intensity of the peaks at 36.08° and 54.32° became stronger after etching for 8h, which further verified the formation of the Zn(OH)_2_ coating. After modification with PFOA, no new diffraction peaks appear due to the amorphous structure of PFOA.

### 3.2 Wetting properties of different samples

**FIGURE 4:**
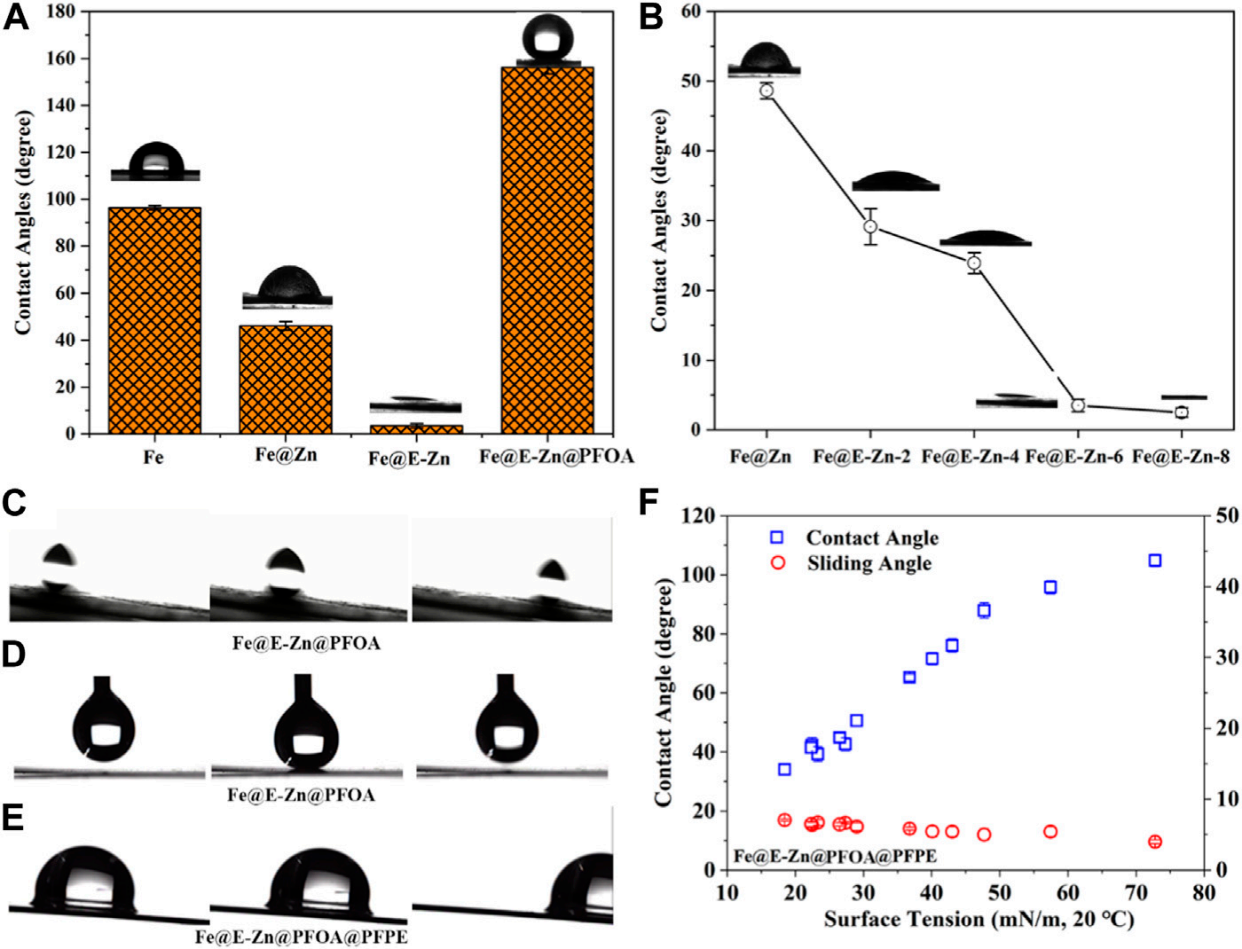
**(A)** Water contact angles of different samples. **(B)** Water contact angles of Fe@E-Zn after vapor-etching for different times. **(C)** Contact angles and sliding angles of different liquids on the Fe@E-Zn@PFOA@PFPE. **(D–E)** Water rolling process and dynamic contact process of water droplet on the Fe@E-Zn@PFOA. **(F)** Water sliding process on the Fe@E-Zn@PFOA@PFPE.

As everyone knows, the interfacial wetting phenomenon is mainly determined by its surface roughness and chemical composition. Thus, by regulating their structure and chemical components, the wettability of different samples was tested as shown in Figure 4. The original Fe plate is hydrophobic and the water contact angle is close to 96°. The Fe@Zn changed to hydrophilicity with a water contact angle of nearly 55°. Further etching by vapor, the Fe@E-Zn-6h with massive porous structure and hydroxyl groups changed superhydrophilicity (Figure 4A). Besides, the influences of the etching time on their wettability was investigated as demonstrated in Figure 4B. The contact angles of Fe@E-Zn−2h and Fe@E-Zn-4h are nearly 32° and 24°, respectively. For the Fe@E-Zn-6h, there is a small contact angle of nearly 5°. Increasing the etching time to 8h, the obtained Fe@E-Zn-8h performed superhydrophilicity, with a contact angle of nearly 0°. Meanwhile, the influences of etching time on the oil spreading time and maximum oil absorption were investigated as shown in Supplementary Figure S6. With increasing etching time, the spreading time of 20 uL PFPE oil on the samples gradually decreased from 2.2 s to 0.8 s. Moreover, the maximum oil absorption of the sample increased from 15.6 ± 0.45 mg/cm^2^ to 18.2 ± 0.65 mg/cm^2^. It can be attributed that with prolonging etching time, the Honeycomb-like porous structure became more complete and rougher which enhanced hydrophilicity, oleophilicity, and oil storage properties. Furthermore, after post-modification with the PFOA, the obtained Fe@E-Zn@PFOA performed superhydrophobicity with a water contact angle of 152° ± 3.5°, respectively. Meanwhile, water droplets could easily roll off the surface of the Fe@E-Zn@PFOA with a tilt angle of nearly 5°, further verifying their superior repellency to water with low adhesive forces (Figure 4C). Also, the Fe@E-Zn@PFOA exhibited superior water repellency, on which water droplets could lift easily without leaving any residues, as shown in Figure 4D. After the Fe@E-Zn@PFOA infused with PFPE lubricant, the formed Fe@E-Zn@PFOA@PFPE showed omniphobicity with sliding angles lower than 10° to various liquids with surface energy in the range from 18.43 mN/m to 72.8 mN/m, as shown in Figure 4F. The contact angles of various liquids on the omniphobic surface increased with the increase of surface tension. Figure 4E shows the time-sequenced images of water droplets sliding off the inclined Fe@E-Zn@PFOA@PFPE.

The evaporation and loss of lubricant on the Fe@E-Zn@PFOA@PFPE generally affects their surficial properties. The stability of the prepared Fe@E-Zn@PFOA@PFPE was evaluated by testing their water sliding angle (WSA) and oil sliding angle (OSA) after treatment by high/low-temperature tests, UV irradiation tests, and water washing tests, as demonstrated in Supplementary Figure S7. As shown in Supplementary Figure S7A, S7B, the WSA and OSA of Fe@E-Zn@PFOA@PFPE showed no obvious alteration after being placed in an oven at 100 °C and a refrigerator at −10 °C for 30 h, respectively. Moreover, after 30 h of ultraviolet irradiation, the WSA and OSA of Fe@E-Zn@PFOA@PFPE changed little, as shown in Supplementary Figure S7C, indicating that the slippery surface could maintain liquid repellency after ultraviolet irradiation. In the water washing experiment, after directly washing for 5 cycles using 50 mL water, the WSA and OSA of Fe@E-Zn@PFOA@PFPE were measured. After washing 25 times, the Fe@E-Zn@PFOA@PFPE maintained stable superior liquid repellency. Thus, the Fe@E-Zn@PFOA@PFPE performed good stability against various harsh conditions to high/low temperatures, UV irradiation, and water washing tests.

### 3.3 Anti-corrosion property of the samples

**FIGURE 5:**
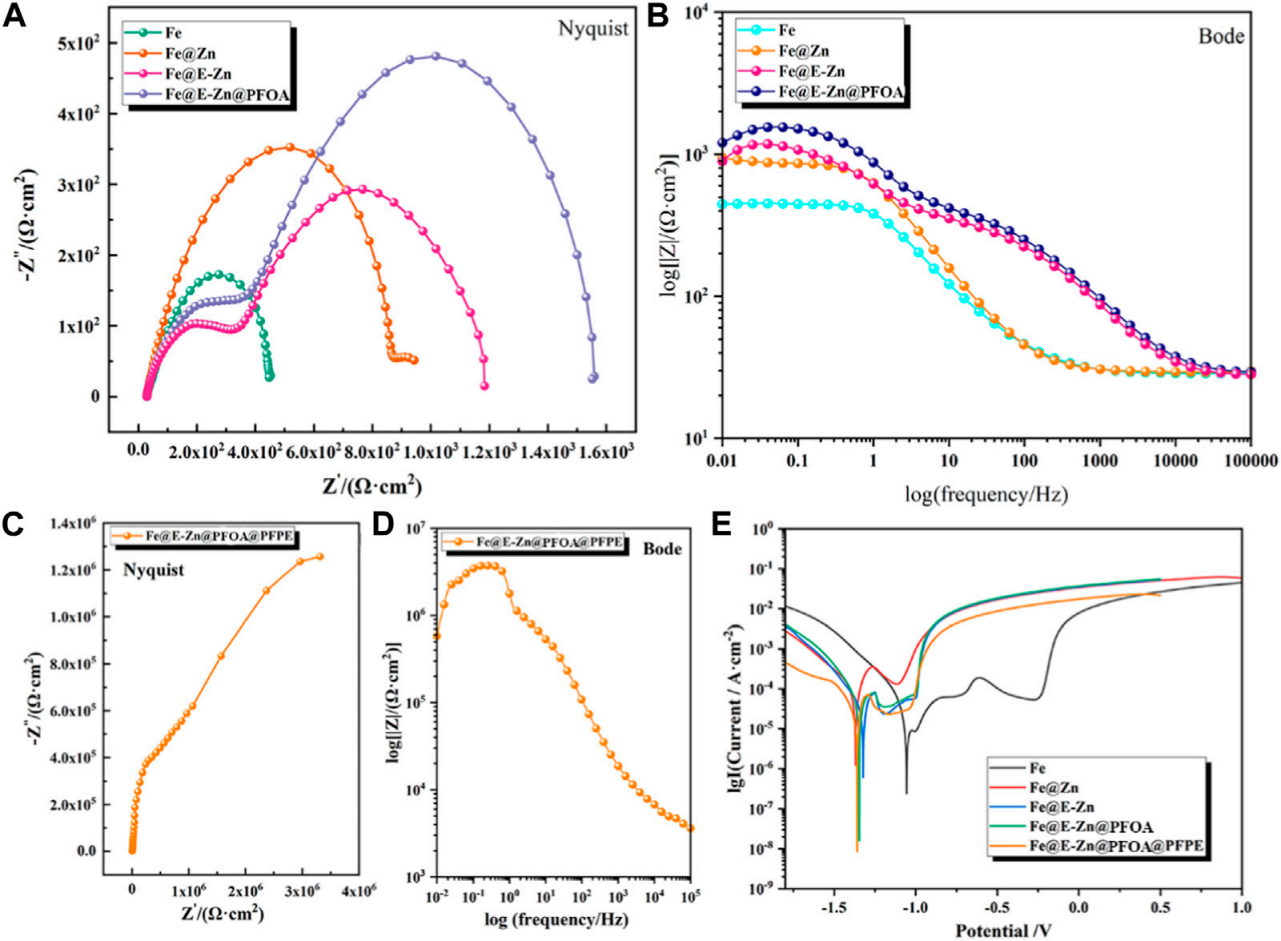
Anti-corrosion property of the different samples. Nyquist plots **(A,C)**, Bode plots **(B,D)**, and Tafel plots **(E)** of the different samples.

By virtue of its superior water-repellency properties, the samples showed good anti-corrosion properties. The corrosion resistance of different samples is evaluated by Electrochemical Impedance Spectroscopy (EIS) and polarization measurements, and the corresponding results are shown in Figure 5. The Nyquist and Bode plots of the Fe, Fe@Zn, Fe@E-Zn, and Fe@E-Zn@PFOA are demonstrated in Figures 5A, B, respectively. In the Nyquist diagram, the semicircular diameter of the capacitor ring related to charge transfer resistance (R_ct_) represents the impedance of the samples. The larger the semicircular diameter, the higher the R_ct_, and the stronger the corrosion resistance of the corresponding sample. Figure 5A is the Nyquist curve of different samples at 3.5wt %. From the semicircular diameter, the anti-corrosion property is as listed: Fe@E-Zn@PFOA > Fe@E-Zn > Fe@Zn > Fe. Meanwhile, for the Bode spectra, the impedance measured at the low-frequency impedance modulus (|Z| = 0.01 Hz) can also express the corrosion resistance of the coating. As demonstrated in Figure 5B, the impedance of Fe@Zn is 850 Ω cm^2^, which is larger than the pure Fe plate (446 Ω cm^2^). After etching, the impedance of the prepared Fe@E-Zn changes to 946 Ω cm^2^. After further post-modification with the PFOA, the impedance of the Fe@E-Zn@PFOA is nearly 1240 Ω cm^2^, which is further increased 1 times compared with the pure Fe because of the air pocket layer trapped between the substrate and solution. As for the Fe@E-Zn@PFOA@PFPE, the Nyquist and Bode plots are listed in Figures 5C, D, respectively. Significantly, the impedance of the Fe@E-Zn@PFOA@PFPE reaches 5.8*10^8^ Ω cm^2^ at lower frequencies, which is 2 orders of magnitude larger than the Fe@E-Zn@PFOA. The superior insulating property of the Fe@E-Zn@PFOA@PFPE is attributed to the lubricant fully covering the whole surface and blocking the electron transfer. In the polarization curves, the higher corrosion current density (i_corr_) represents weak corrosion resistance. As shown in Figure 5E, the i_corr_ of the pristine Fe is nearly 2.47 × 10^−7^ A cm^−2^. As for the Fe@Zn surface, its i_corr_ is nearly 1.25 × 10^−6^ A cm^−2^ due to its high metal activity of zinc coating. Further to etching by vapor, the i_corr_ of Fe@E-Zn is 6.25 × 10^−7^ A cm^−2^, which is still higher than the pure Fe plate. As for the superhydrophobic Fe@E-Zn@PFOA surface, the i_corr_ changes to 1.67 × 10^−8^ A cm^−2^, which is one order of magnitude lower than that of the Fe plate, indicating an enhanced corrosion resistance for the superhydrophobic treatment. Moreover, the lubricant-infused Fe@E-Zn@PFOA@PFPE performs superior corrosion resistance with an i_corr_ of 8.25 × 10^−9^ A cm^−2^, which is in agreement with the EIS results. These results further imply that the hydrophobic treatment and lubricant-infused treatment can greatly improve the anti-corrosion capability of the Fe plate.

## Conclusion

In summary, a Zn coating with a porous honeycomb-like structure was fabricated on the Fe plate by vapor etching-assisted electroplating in deep eutectic solvent. Notably, the simple and environment-friendly water vapor etching process could controllably form a rough petaliform structure. Further to post-modification with PFOA and infused PFPE lubricant, the superhydrophobic Fe@E-Zn@PFOA and omniphobic Fe@E-Zn@PFOA@PFPE surfaces were prepared. The regulation of the morphological structure and chemical compositions resulted in the variation of their wettability and liquid-repellency. Significantly, the lubricant-infused surfaces showed excellent corrosion resistance, which is ascribed to the water-repellent lubricant completely covering the hydrophobic modified porous coating. The proposed lubricant-infused surface is anticipated to play an important role in expanding practical applications in antifouling and anti-corrosion areas.

## Data Availability

The original contributions presented in the study are included in the article/Supplementary Material, further inquiries can be directed to the corresponding authors.
