# Supplementary material for: Vapor-etching honeycomb-like zinc plating layer for constructing anti-corrosion lubricant-infused surfaces
Source: Front Chem. 2023 Sep 28;11:1273674. doi: 10.3389/fchem.2023.1273674 (PMC10568014; doi:10.3389/fchem.2023.1273674)
Supplement: Supplementary file 1 [file Table1.docx]

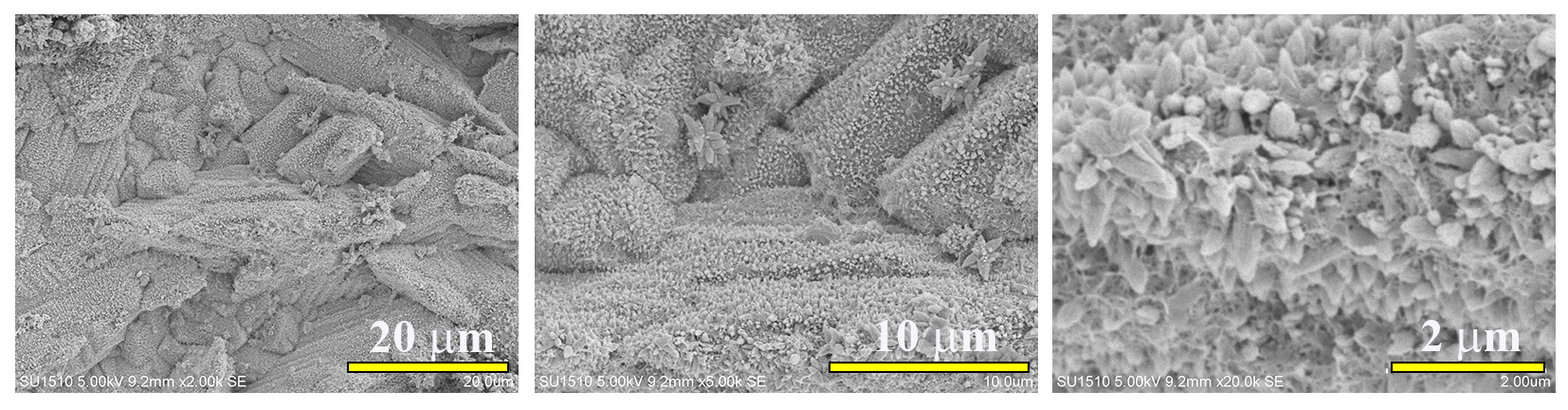


Figure S1. SEM images of the back side of Fe@E-Zn.


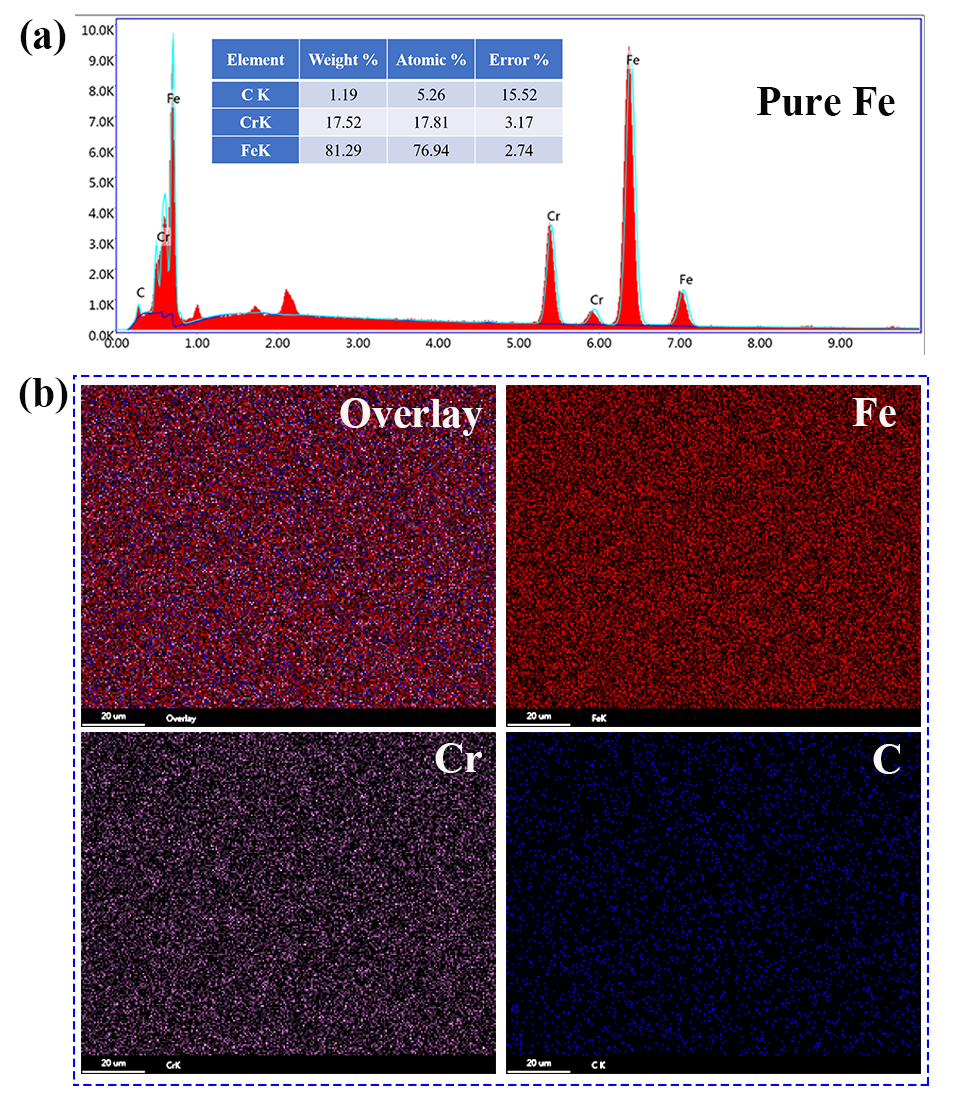


Figure S2. EDS spectra (a) and elements’ mappings (b) of the pure Fe.


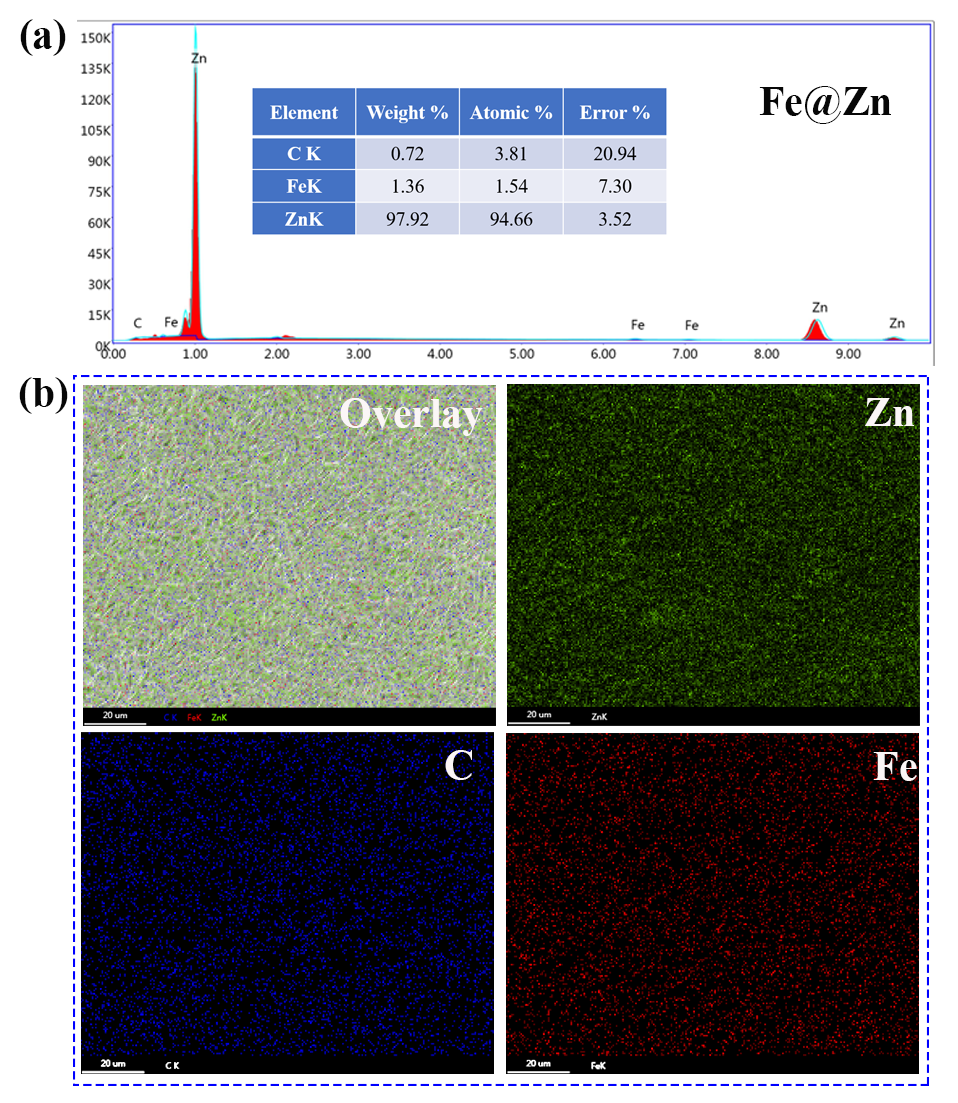


Figure S3. EDS spectra (a) and elements’ mappings (b) of the pure Fe@Zn.


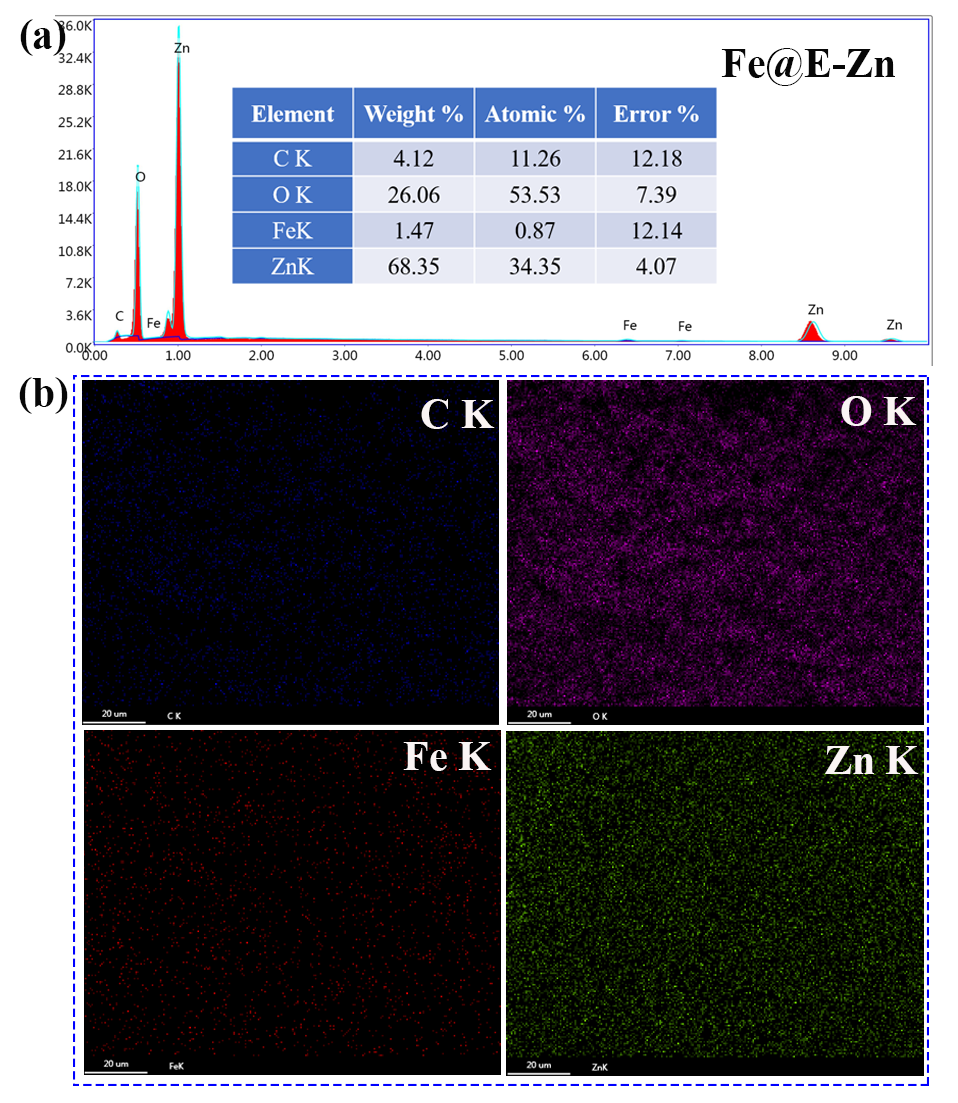


Figure S4 EDS spectra (a) and elements’ mappings (b) of the pure Fe@E-Zn.


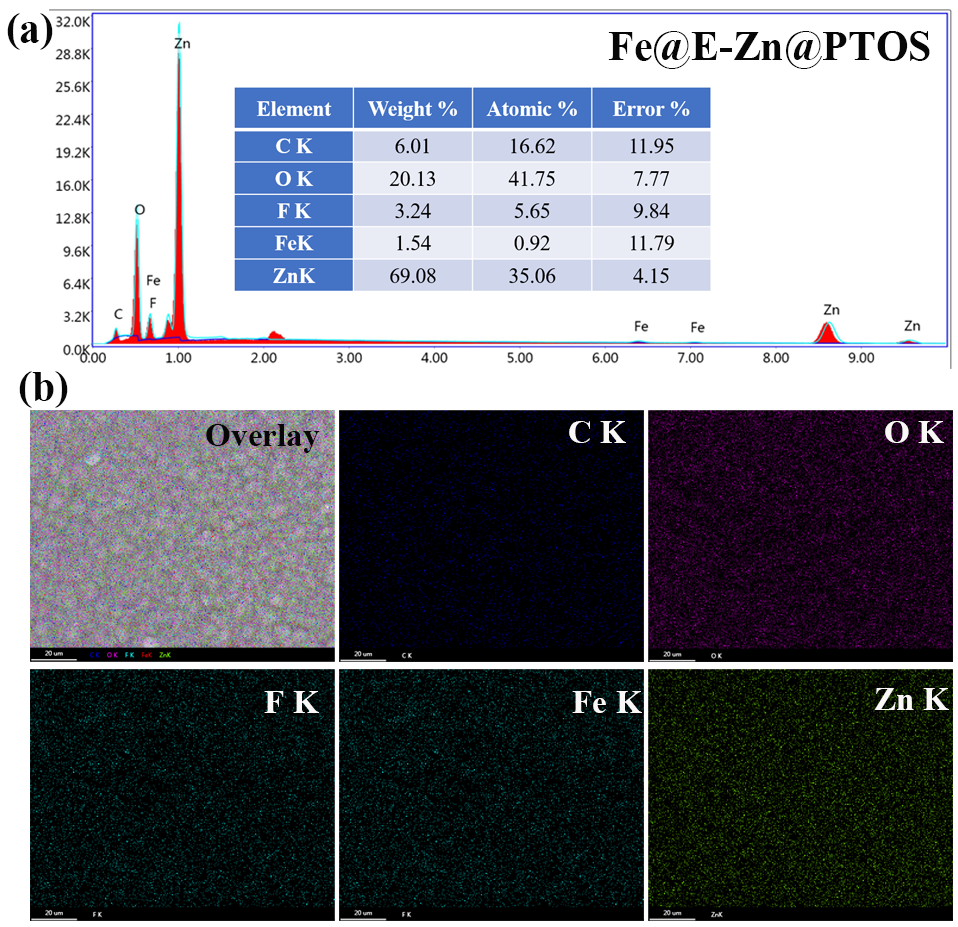


Figure S5 EDS spectra (a) and elements’ mappings (b) of the pure Fe@E-Zn@PTOS.





Figure S6 Oil spreading time (PFPE, 20 uL) and maximum oil absorption of Fe@E-Zn after vapor-etching for different times.


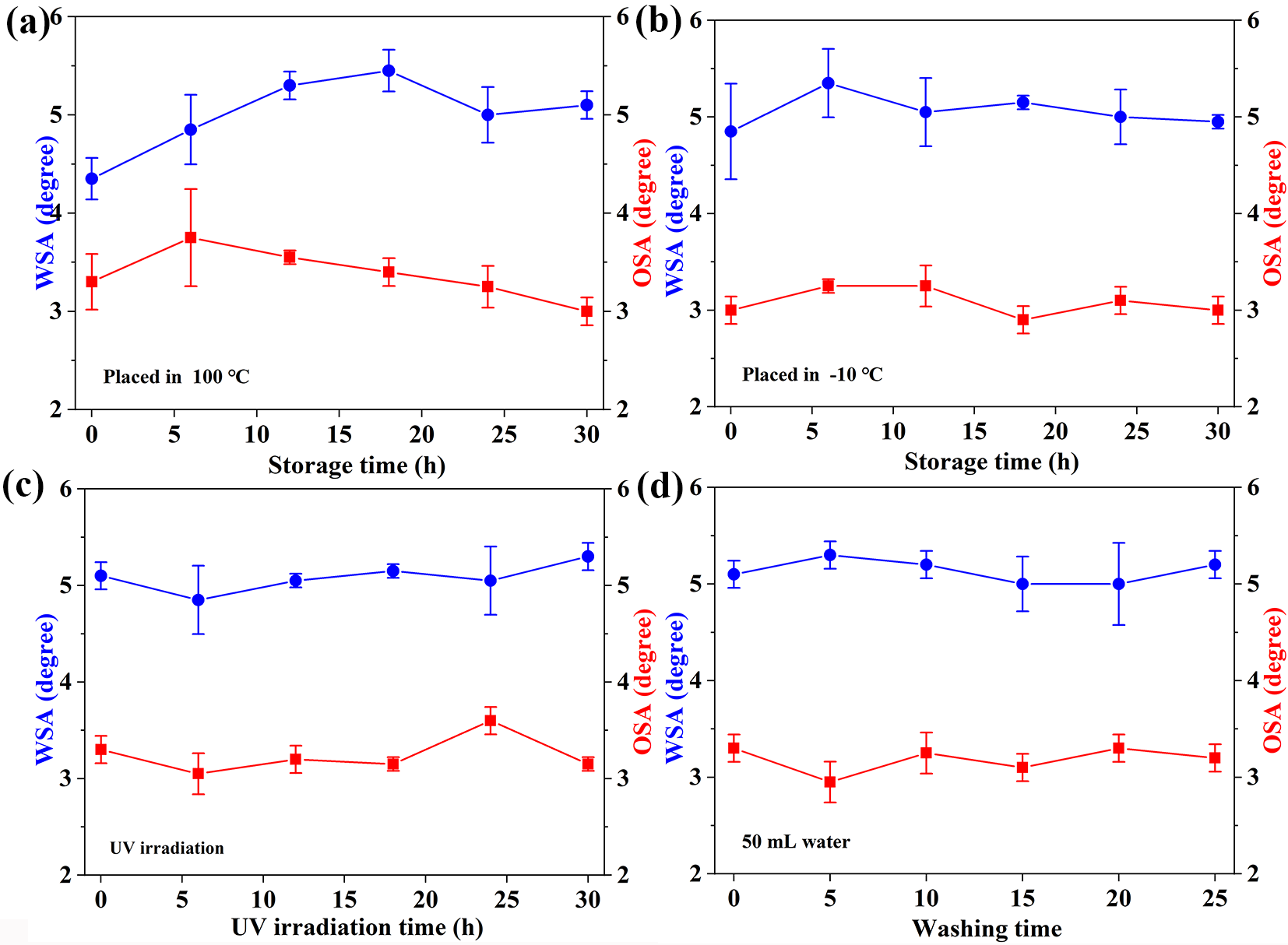


Figure S7 Water sliding angle (WSA, water, 10 uL) and oil sliding angle (OSA, Chloroform, 10 uL) of the Fe@E-Zn@PFOA@PFPE after different treatments including storage in high temperature (100 ℃)，low temperature (-10 ℃), UV irradiation, and water washing for different times.
